# Left Bundle Branch Area Pacing Contributes to a Greater Acute Blood Pressure Reduction Compared to Right Ventricular Pacing

**DOI:** 10.31083/j.rcm2412372

**Published:** 2023-12-27

**Authors:** Sijin Wu, Wenzhao Lu, Zhongli Chen, Qingyun Hu, Yao Li, Yuan Gao, Wei Wang, Ying Wu, Ruohan Chen, Yan Dai, Keping Chen, Shu Zhang

**Affiliations:** ^1^State Key Laboratory of Cardiovascular Disease, Arrhythmia Center, Fuwai Hospital, National Center for Cardiovascular Diseases, Chinese Academy of Medical Sciences & Peking Union Medical College, 100037 Beijing, China

**Keywords:** left bundle branch area pacing, right ventricular pacing, physiological pacing, blood pressure control, propensity score-matching

## Abstract

**Background:**

Several previous studies have explored the potential arterial blood pressure (BP) changes in patients undergoing right ventricular pacing (RVP), however, the relationship between left bundle branch area pacing (LBBAP) and BP variations remains unknown. This study aimed to examine the acute BP variations following LBBAP and RVP implantation in patients with bradycardia.

**Methods:**

We conducted a single-center retrospective study including all patients who underwent de-novo dual-chamber pacemaker implantation between January 2019 and June 2021. Patients were divided into two groups, LBBAP and RVP, and propensity score-matching (PSM) was used to balance confounding factors. Three time periods were defined according to the timing of the implant: baseline (within 24 hours before implantation), hyper-acute period (0–24 hours post-implantation), and acute period (24–48 hours post-implantation). BP was measured at least three times per period using an arm pressure cuff and then averaged for analysis, which allowed us to determine the acute impact of pacemaker implantation on BP.

**Results:**

From a cohort of 898 patients, 193 LBBAP receivers were matched to 193 RVP receivers. A significant decrease in systolic BP (SBP) after the implantation was observed in the study cohort, from baseline 137.3 ± 9.2 mmHg to the acute period of 127.7 ± 9.4 mmHg (*p <* 0.001). The LBBAP group exhibited a greater SBP reduction than the RVP group (Δ11.6 ± 6.2 mmHg vs. Δ7.6 ± 5.8 mmHg, *p* < 0.001). In further subgroup analysis, LBBAP receivers who had high baseline SBP (*p* < 0.001) and those without using anti-hypertensive drugs (*p* = 0.045) appeared to have a higher magnitude of SBP reduction.

**Conclusions:**

Permanent pacemaker implantation may contribute to an acute decrease in systolic BP, which was more pronounced in LBBAP receivers. Future experimental and clinical investigations are necessary to explore the underlying mechanisms and the long-term hemodynamic effects of LBBAP versus RVP.

## 1. Introduction

Cardiac pacing is the primary treatment for symptomatic bradycardia or high-grade atrioventricular block [1]. In clinical practice, right ventricular pacing (RVP) has been a well-established technique for decades. Accumulated evidence indicates that chronic RVP can cause dyssynchronous left ventricular electrical activation and consequently results in deleterious impacts on cardiac function [2, 3, 4]. This is particularly true for patients with a high percentage of ventricular pacing (VP%), typically defined as VP% >40%. Left bundle branch area pacing (LBBAP), an emerging physiological pacing modality, has been introduced in recent years [5]. By directly stimulating the cardiac conduction system, LBBAP can preserve intraventricular and interventricular electrical and mechanical synchrony [6, 7]. Multiple studies have demonstrated the clinical superiority of LBBAP over conventional RVP, including improvements in all-cause mortality, reductions in heart failure hospitalization, and a decreased risk of atrial fibrillation [8, 9, 10].

Blood pressure (BP) is a useful hemodynamic indicator that is dynamically modulated by both the autonomic nervous system (ANS, sympathetic and vagal reflex) and neurohumoral factors (circulating catecholamines, neuropeptides, rein-angiotensin-aldosterone-system). Several studies have reported that patients with chronic bradycardia may experience elevated systolic blood pressure (SBP) [11, 12, 13], which can be attributed to an increased myocardial contraction force (Frank-Starling mechanism) and excessive sympathetic activation secondary to bradycardia. Vice versa, elevated SBP can also lead to bradycardia via baroreflex. An unexpected phenomenon commonly observed is that RVP receivers can undergo BP variations after the implantation, which is termed ‘pacemaker syndrome’ [14, 15]. It refers to a complex of symptoms, consisting of fatigue, palpitations, and shortness of breath, which are related to the adverse hemodynamic and electrophysiological consequences of ventricular pacing. Several potential causes have been linked to the occurrence of pacemaker syndrome, which include retrograde atrial conduction, disruption of regular atrial synchrony at a normal atrioventricular (AV) interval, and insufficient cardiac rate response [14]. Previous studies proposed that pacing might participate in hemodynamic abnormalities by adversely impacting cardiac autonomic baroreflex function, particularly in non-physiological pacing [16, 17]. However, the exact hemodynamic effect of pacing has received little attention in the literature, and no studies have yet focused on the BP variations associated with novel physiological pacing.

We hypothesized that the magnitude of BP variations in ventricular-paced individuals might differ depending on the pacing modalities. Given that LBBAP produces physiological cardiac contractile outcomes of the left ventricle due to the native conduction system, it is likely to confer more favorable hemodynamic effects over less physiological RVP. To test this hypothesis, we examined the BP values pre- and post-implantation in patients who received either LBBAP or RVP.

## 2. Materials and Methods

### 2.1 Study Design and Population

This study was a single-center, retrospective, observational study. We consecutively enrolled 1001 adult patients (≥18 years old) who underwent *de-novo* dual-chamber permanent pacemaker implantation (PPI) at Fuwai Hospital (Beijing, China) between 1 January 2019 and 30 June 2021. The decision for pacemaker treatment was made according to Class I or II guideline recommendations [1]. To minimize the effect of confounding factors on hemodynamics, we excluded patients if they: (i) underwent cardiac surgery within 1 month prior to PPI; (ii) received additional interventional procedures within 2 weeks before implantation (coronary angiography, catheter ablation of tachyarrhythmias, transcatheter aortic valve replacement); (iii) received continuous infusions of vasoactive drugs within 24 hours before implantation (adrenaline, isoprenaline, norepinephrine, dopamine). In cases of isoproterenol non-response, after diagnosing atrioventricular block (AVB), we typically wait for an individualized period to observe the patient’s condition and symptom progression; (iv) experienced early post-implantation complications within 2 days, such as lead dislodgement, elevated capture threshold (>5 V/0.4 ms), pneumothorax, and infection. Patients were divided into two groups by pacing modality, LBBAP and RVP, which were determined by the operators based on clinical practice. This study conformed with the Declaration of Helsinki and was approved by the Ethics Committee of Fuwai hospital (No.2019-1149). All participants provided written informed consent for pacemaker implantation and use of data for clinical investigation.

### 2.2 Pacing Procedure and Device Programming

The LBBAP procedure involves capturing left bundle branch (LBB) via a transventricular-septal approach, as previously described in detail [18]. Briefly, a delivery C315HIS fixed curve sheath with a SelectSecure 3830 pacing lead (Medtronic Inc., Minneapolis, MN, USA) was first positioned in the His bundle region. Then we rotated the sheath to place the lead perpendicularly against the interventricular septum (IVS) and gently advanced the lead helix toward the left side of the septum. The lead was further advanced until LBBAP criteria were achieved. A successful LBBAP procedure was defined as [19]: (i) the paced QRS morphology showed a pattern of right bundle branch conduction delay (RBBD) in lead V1/2 on electrocardiogram (ECG); (ii) an LBB potential was recorded or a shortened peak left ventricular activation time (LVAT) in lead V5/6 was present. RVP was performed using an active fixation lead, which involved implanting the right ventricular lead at the right apex or ventricular septum. The atrial lead was inserted into the right atrial appendage.

As part of our routine practice, we performed device programming at implantation and within 48 hours after pacemaker implantation. An individualized AV delay was programmed according to the intrinsic AV conduction and bundle branch block. To prevent unnecessary ventricular pacing, the automatic AV search algorithm was routinely enabled in patients with intact AV conduction. Multiple parameters were assessed and recorded, including pacing electrical parameters (lead impedance, capture threshold, and R-wave amplitude), sensing amplitude, VP%, and percentage of atrial pacing (AP%).

### 2.3 Clinical Outcomes and Measurements

The primary outcome of this study was the acute changes in SBP shortly after pacemaker implantation. According to the diagnostic criteria for hypertension established by the International Society of Hypertension (ISH) in 2020 [20], hypertension would be diagnosed when a person’s SBP was ≥140 mmHg and/or their diastolic blood pressure (DBP) was ≥90 mmHg. Considering the dynamic nature of blood pressure, three time periods were defined according to the implant timing: baseline (within 24 hours before implantation), hyper-acute period (0–24 hours post-implantation), and acute period (24–48 hours post-implantation). In our unit, all patients undergoing PPI were connected to remote cardiac monitoring during the periprocedural period. BP was measured using an arm pressure cuff at least 3 times per period, along with heart rate (HR). Patients were allowed to rest for 5 minutes before measurements. During blood pressure measurement, patients usually sit in a comfortable position with their arm at heart level and feet flat on the floor. The cuff was then placed around the upper arm, ensuring a snug but not overly tight fit. To ensure accurate measurements, we selected appropriately sized cuffs based on the patient’s arm circumference. Typically, the cuff must cover 75–100% of the individuals arm circumference. All measurements were taken with a fixed arm in an awake state and the values were averaged for analysis. Given the potential for wound pain and anxiety to cause fluctuations in BP and HR, only the values recorded 12–24 hours after implantation were analyzed in the hyper-acute period. All BP and HR data were documented in the patient chart or nursing sheets via the electronic medical system.

### 2.4 Data Collection

All data collection occurred while patients were admitted into the hospital for pacemaker implantation and before patients were discharged post-implantation. We collected various patient data including demographics, vital signs at each period, cardiovascular comorbidities, antihypertensive drugs (AHDs), ECG and Holter parameters, echocardiography parameters, and periprocedural information. All data were extracted from our hospital’s electronic medical record system. To ensure data accuracy, approximately 5% of medical records were randomly selected and reviewed during the preliminary extraction phase.

### 2.5 Statistical Analysis

Continuous variables were expressed as the mean ± standard deviation (SD) or the median (interquartile range). The Student *t*-test or Wilcoxon rank-sum test was used for comparing continuous variables. Categorical values were presented as counts with percentages and were compared using Pearson’s χ^2^ test or Fisher’s exact test.

To minimize confounding bias, we employed propensity score-matching (PSM) [21], which involved estimating the propensity score (PS) using a logistic regression model containing all covariates listed in Table 1. LBBAP and RVP groups were matched at a 1:1 ratio with a 4-digit nearest neighbor algorithm within a caliper of 0.2. In the PSM cohort, repeated measures analysis of variance (ANOVA) was applied to examine the overall SBP changes across all three time points, and Tukey’s method was used for multiple comparisons. An independent sample *t*-test was used to compare the LBBAP and RVP groups. We performed subgroup analyses within each group to explore potential factors that could influence variations in BP. Subgroup categories were specified by baseline SBP, hypertension status, and use of AHDs. A Pearson correlation analysis was conducted to explore the linear relationship between numerical variables.

**Table 1.** **Baseline characteristics of the study population in both the entire cohort and the propensity score-matching cohort**.

		Entire cohort			Propensity-score matching cohort		
		LBBAP	RVP	*p *value	LBBAP	RVP	*p *value
		(n = 209)	(n = 689)		(n = 193 )	(n = 193)	
Age, yrs		61.7 ± 13.4	67.5 ± 12.8	<0.001	62.4 ± 12.8	63.7 ± 16.3	0.391
Female, n (%)		103 (49.3)	381 (55.3)	0.147	99 (51.3)	91 (47.2)	0.476
HR, bpm		55.9 ± 12.9	61.7 ± 12.5	<0.001	56.8 ± 12.9	57.7 ± 12.9	0.466
24 h mean ${\mathrm{HR}}^{a}$, bpm		55.3 ± 9.7	58.8 ± 11.7	<0.001	55.5 ± 9.8	56.3 ± 10.1	0.450
SBP, mmHg		137.1 ± 9.4	135.4 ± 8.7	0.019	136.8 ± 9.1	137.7 ± 9.4	0.322
DBP, mmHg		73.1 ± 7.2	73.2 ± 6.8	0.928	73.3 ± 7.1	73.8 ± 7.4	0.545
LVEF, %		62.0 ± 5.9	62.6 ± 4.7	0.180	62.2 ± 5.9	61.9 ± 4.9	0.620
LVEDD, mm		48.2 ± 5.3	47.4 ± 5.2	0.033	48.1 ± 5.1	48.4 ± 6.1	0.646
Pacemaker indication, n (%)				<0.001			0.918
	SND	83 (39.7)	548 (79.5)		83 (43.0)	81 (42.0)	
	AVB	126 (60.3)	141 (20.5)		110 (57.0)	112 (58.0)	
Intrinsic ${\mathrm{rhythm}}^{b}$, n (%)				<0.001			0.830
	Sinus	179 (85.6)	608 (88.2)		168 (87.0)	173 (89.6)	
	AF	3 (1.4)	32 (4.6)		3 (1.6)	3 (1.6)	
	Escape	23 (11.0)	24 (3.5)		18 (9.3)	13 (6.7)	
	Temporary pacing	4 (1.9)	25 (3.6)		4 (2.1)	4 (2.1)	
${\mathrm{Comorbidities}}^{c}$							
	Hypertension, n (%)	130 (62.2)	423 (61.4)	0.897	119 (61.7)	115 (59.6)	0.755
	Dyslipidemia, n (%)	96 (45.9)	338 (49.1)	0.476	88 (45.6)	92 (47.7)	0.760
	Coronary artery disease, n (%)	56 (26.8)	206 (29.9)	0.437	52 (26.9)	56 (29.0)	0.734
	AF/AFL, n (%)	31 (14.8)	253 (36.7)	<0.001	31 (16.1)	27 (14.0)	0.669
Antihypertensive drugs							
	Beta-Blocker, n (%)	82 (39.2)	271 (39.3)	1.000	75 (38.9)	75 (38.9)	1.000
	ACEi/ARB, n (%)	89 (42.6)	211 (30.6)	0.002	78 (40.4)	75 (38.9)	0.835
	CCB, n (%)	77 (36.8)	203 (29.5)	0.053	72 (37.3)	65 (33.7)	0.523
	Diuretic, n (%)	42 (20.1)	69 (10.0)	<0.001	34 (17.6)	31 (16.1)	0.786

^a^ Defined as the 24-hour average heart rate recorded by Holter. ^b^ Defined as intrinsic rhythm at implant. ^c^ Defined as a history of such a disease. Abbreviations: ACEi/ARB, angiotensin-converting enzyme inhibitor/angiotensin receptor blocker; AF/AFL, atrial fibrillation/atrial flutter; AVB, atrioventricular block; CCB, calcium channel blockers; DBP, diastolic blood pressure; HR, heart rate; LVEDD, left ventricular end-diastolic dimension; LVEF, left ventricular ejection fraction; SBP, systolic blood pressure; SND, sinus node dysfunction; LBBAP, left bundle branch area pacing; RVP, right ventricular pacing.

To maximize statistical power and minimize bias that might occur if small proportions of missing data were excluded from analyses, we applied a multiple imputation with chained equations to impute missing values [22]. All statistical analyses were performed using R (version 4.1.2, 2021-11-01, Boston, MA, USA) and a two-sided *p* < 0.05 was considered significant.

## 3. Results

### 3.1 Baseline Characteristics of the Study Population

**Fig. 1.**
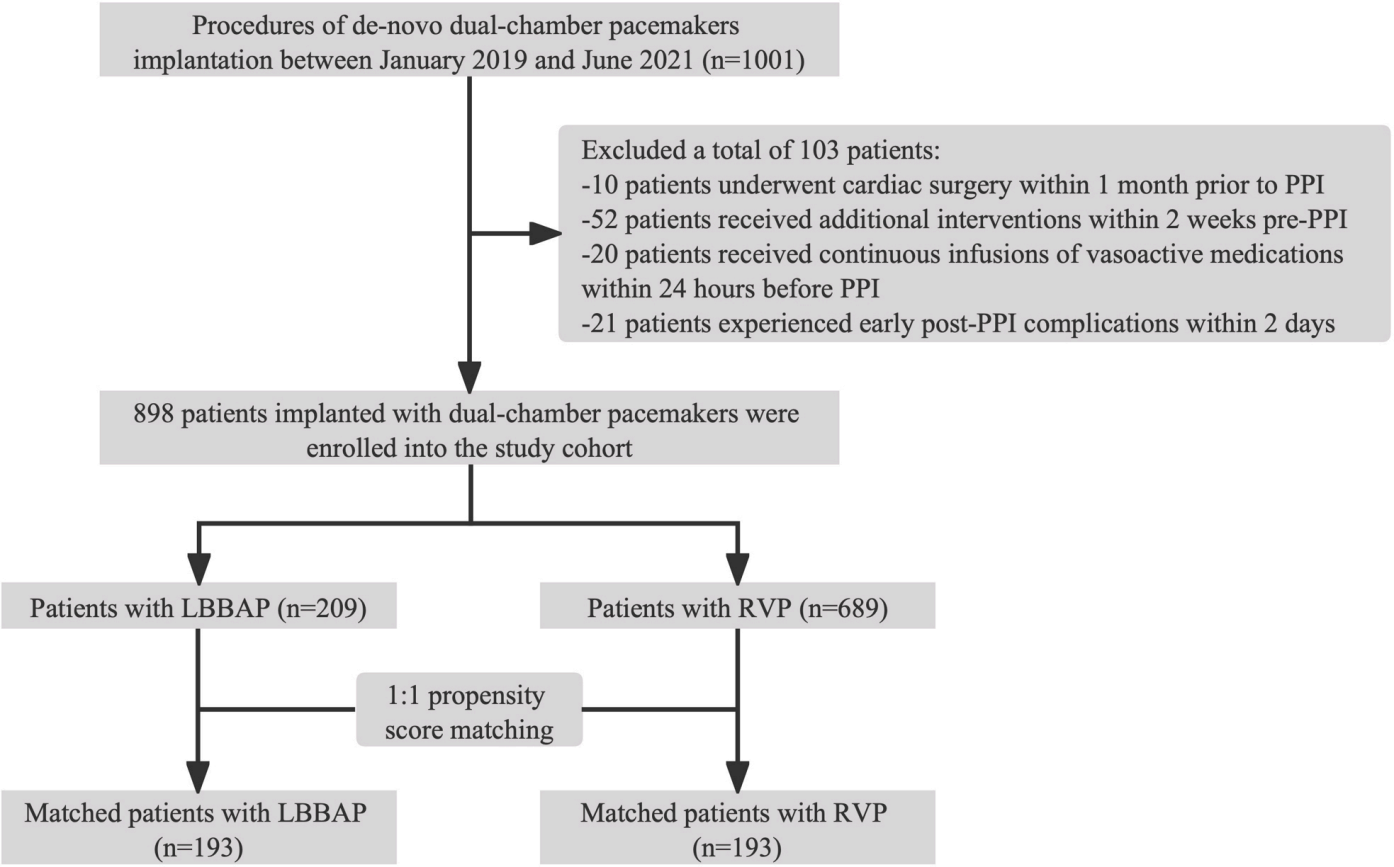
**Flowchart of patients selection in the cohort**. Abbreviations: LBBAP, left bundle branch area pacing; PPI, permanent pacemaker implantation; RVP, right ventricular pacing.

In the entire cohort, LBBAP was successfully achieved in 209 patients, whereas 689 patients received RVP, of which, 386 propensity-matched patients (193 LBBAP receivers; 193 RVP receivers) were extracted to serve as the PSM cohort (Fig. 1). Before PSM, the LBBAP group was significantly younger than in RVP (*p <* 0.001) and had a lower baseline HR and SBP *(p <* 0.001), fewer instances of atrial fibrillation/atrial flutter history (*p* < 0.001), and more prescriptions for AHDs. The LBBAP group had a higher proportion of AVB cases compared to the RVP group (60.3% vs. 20.5%, *p* < 0.001) and significant differences in intrinsic rhythm at implant between the two groups (*p* < 0.001). After 1:1 propensity matching, the two groups were comparable for baseline data. The baseline characteristics of the study population before and after PSM were summarized in Table 1.

### 3.2 ECG and Pacing Parameters

The baseline QRS duration (QRSd) and intrinsic QRS morphology were found to be similar between the LBBAP and RVP groups (*p *= 0.937, *p* = 0.283, respectively). However, LBBAP resulted in a relatively narrower paced QRS complex compared to RVP (117.4 ± 18.4 ms vs. 151.7 ± 13.8 ms, *p* < 0.001), and a higher pacing threshold (*p* < 0.001). There was no significant difference in sensing amplitude (*p* = 0.083) and lead impedance (*p* = 0.145) tested at implant between the two groups, nor in AP% (*p* = 0.650) and VP% (*p *= 0.117). Further details on the ECG and pacing parameters for the PSM cohort can be found in Table 2.

**Table 2.** **ECG and pacing parameters in PSM cohort**.

		LBBAP	RVP	*p* value
Baseline QRS duration, ms		99.9 ± 18.5	100.1 ± 20.7	0.937
Pacing QRS duration, ms		117.4 ± 18.4	151.7 ± 13.8	<0.001
Intrinsic QRS morphology				0.283
	Normal	161	157	
	IVCD	4	11	
	LBBB	7	8	
	RBBB	21	17	
Pacing ${\mathrm{threshold}}^{a}$, V		0.6 ± 0.2	0.5 ± 0.1	<0.001
Sensing amplitude, mV		10.6 ± 4.7	9.8 ± 4.3	0.083
Impedance, ohms		769.3 ± 139.5	792.4 ± 169.0	0.145
Lower rate		60.0 ± 0.7	59.9 ± 0.8	0.739
AP% after ${\mathrm{implant}}^{b}$		10.4 (1.0, 65.9)	20.0 (1.0, 71.6)	0.650
VP% after implant		29.0 (5.5, 99.7)	22.0 (2.1, 98.0)	0.117

^a^ Pacing threshold, sensing amplitude, and impedance were all intraoperative data. ^b^ AP% and VP% were recorded from the first post-operative programmed data. Abbreviations: IVCD, intraventricular conduction delay; LBBB, left bundle branch block; RBBB, right bundle branch block; AP%, percentage of atrial pacing; VP%, percentage of ventricular pacing; PSM, propensity score-matching; LBBAP, left bundle branch area pacing; RVP, right ventricular pacing; ECG, electrocardiogram.

### 3.3 SBP Variations Over Time Periods

**Fig. 2.**
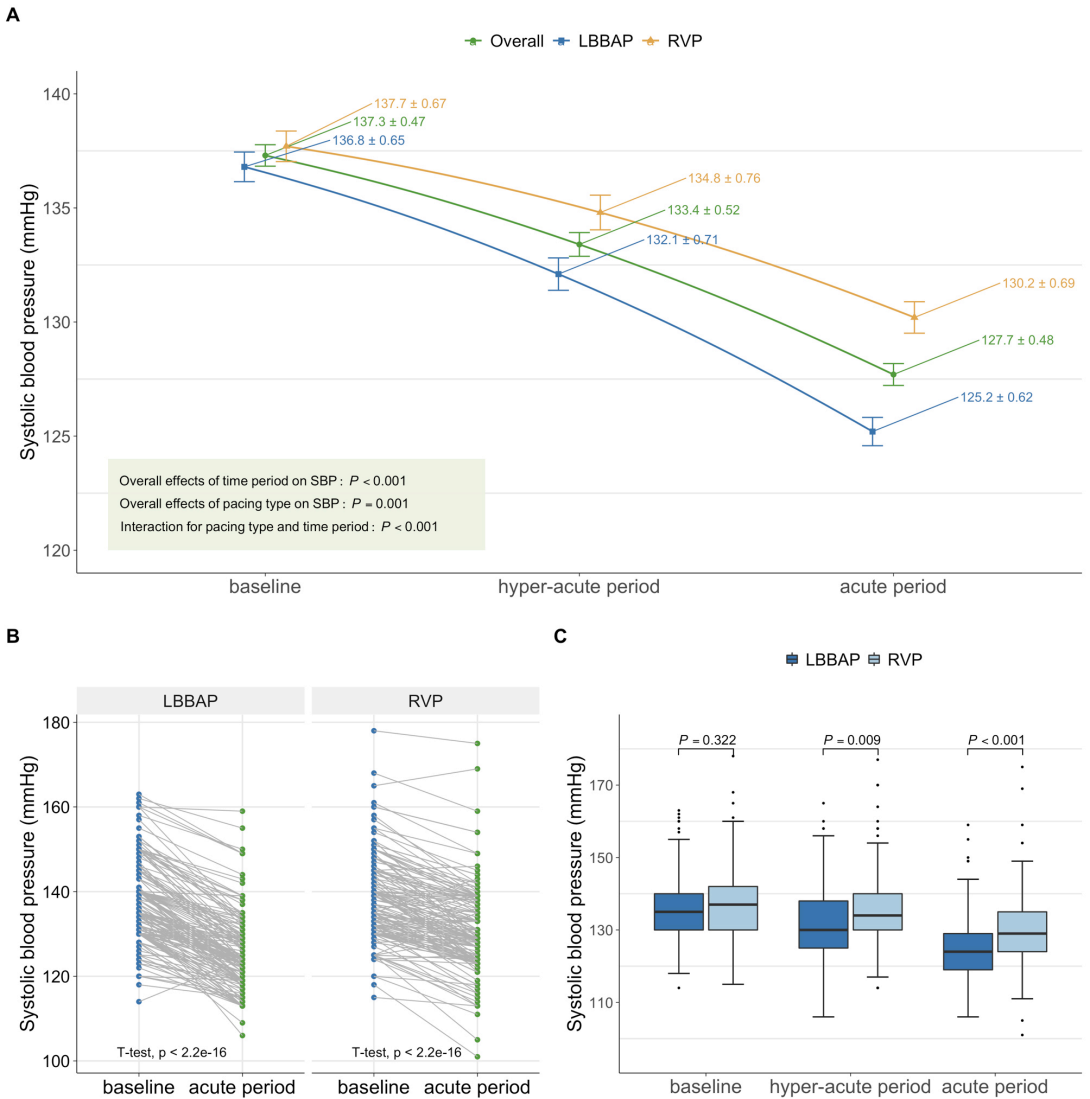
**SBP changes over time periods in the PSM cohort**. (A) SBP changes of the overall population, the LBBAP group, and the RVP group (overall effect was calculated using repetitive measure analysis of variance). The error bar refers to the mean ± standard error (SE). (B) Individual SBP change from baseline to the acute period (*p *values were calculated by paired *t*-test). (C) SBP distributions compared between LBBAP group and RVP group (*p* values were calculated by independent samples *t*-test). Abbreviations: LBBAP, left bundle branch area pacing; PSM, propensity-score matching; RVP, right ventricular pacing; SBP, systolic blood pressure.

A gradual day-over-day reduction in systolic BP after the implantation was observed in the study cohort, from baseline 137.3 ± 9.2 mmHg to the hyper-acute period of 133.4 ± 10.3 mmHg, and then to the acute period of 127.7 ± 9.4 mmHg (*p <* 0.001) (Fig. 2A). Multiple comparisons of SBP across the three timepoints were statistically significant (all adjusted *p* < 0.001). A similar trend was observed in both LBBAP and RVP groups (Fig. 2A). Individual SBP changes compared between baseline and the acute period were displayed in Fig. 2B. Comparisons of SBP between the two groups were provided in Fig. 2C and and Table 3. There was no significant difference in systolic BP between the LBBAP and RVP group at baseline (*p* = 0.322). However, after pacemaker implantation, patients who underwent LBBAP experienced a greater SBP reduction compared to those with RVP. In the hyper-acute period, the LBBAP group had a mean SBP of 132.1 ± 9.9 mmHg, which was significantly lower than the RVP group’s mean SBP of 134.8 ± 10.3 mmHg (mean difference [MD] –2.7; 95% CI –4.8 to –0.7, *p* = 0.009). In the acute period, the mean SBP in the LBBAP group was 125.2 ± 8.5 mmHg, while the RVP group SBP was 130.2 ± 9.4 mmHg in the RVP group (MD –5.0; 95% CI –6.8 to –3.2, *p* < 0.001).

**Table 3.** **SBP values at each time period after pacemaker implantation **.

	Before matching				After matching			
	LBBAP	RVP	Mean difference (95% CI)	*p* value	LBBAP	RVP	Mean difference (95% CI)	*p* value
	(n = 209)	(n = 689)			(n = 193)	(n = 193)		
Mean SBP in hyper-acute period, mmHg	131.4 ± 10.3	132.8 ± 9.8	–1.4 (–3.0, 0.2)	0.090	132.1 ± 9.9	134.8 ± 10.3	–2.7 (–4.8, –0.7)	0.009
Mean SBP in acute period, mmHg	125.0 ± 9.1	127.0 ± 8.7	–2.0 (–3.4, –0.6)	0.005	125.2 ± 8.5	130.2 ± 9.4	–5.0 (–6.8, –3.2)	<0.001
SBP variation from baseline, mmHg	12.1 ± 6.1	8.4 ± 5.9	3.7 (2.7, 4.6)	<0.001	11.6 ± 6.2	7.6 ± 5.8	4.0 (2.9, 5.2)	<0.001

Abbreviations: LBBAP, left bundle branch pacing; RVP, right ventricular pacing; SBP, systolic blood pressure.

Compared to baseline, both the LBBAP and RVP groups showed a reduction in SBP within 24 to 48 hours post-procedure, with the LBBAP group experiencing a significantly greater decrease of 11.6 ± 6.2 mmHg compared to the RVP group of 7.6 ± 5.8 mmHg. The difference in the SBP change between the two groups was found to be 4.0 mmHg (95% CI 2.9 to 5.2, *p* < 0.001), indicating that the LBBAP group had a more pronounced reduction in SBP. This trend was consistent when analyzing data from the entire cohort, with the LBBAP group exhibiting a greater reduction in SBP by 3.7 mmHg compared to the RVP group (95% CI 2.7 to 4.6, *p* < 0.001).

### 3.4 Factors Influencing SBP Changes

**Fig. 3.**
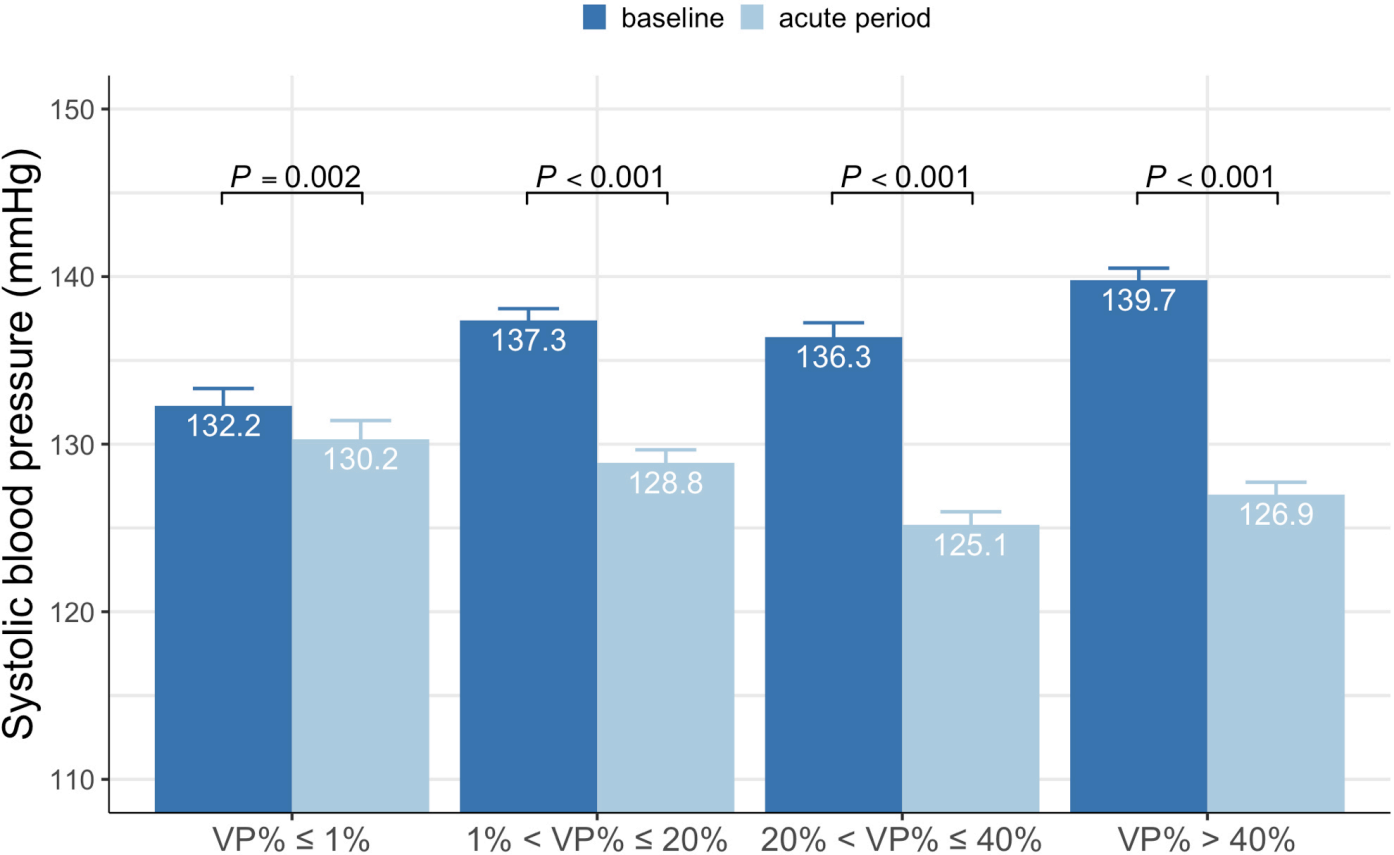
**SBP at baseline and the acute period in different VP% group**. SBP, systolic blood pressure; VP%, percentage of ventricular pacing.

To investigate how pacing affects SBP, we stratified all patients enrolled in the PSM cohort into four groups by VP%: VP% ≤1%, 1% < VP% ≤ 20%, 20% < VP% ≤ 40%, and VP% >40% (with the VP% data following a mimic U-shaped distribution). All groups experienced a decrease in SBP at the acute period compared to their baselines. Patients with VP% >40% had a maximum decrease in SBP, whereas those with VP% ≤1% experienced a minimal decrease (Fig. 3). Further analysis indicated that patients with VP% >40% had a 72.5% percentage of AVB, which was markedly higher than the other groups (**Supplementary Table 1**). In addition, patients with VP% ≤1% were more likely to receive pacemaker implantation for sinus node dysfunction (SND). However, we did not observe a significant difference in SBP change when compared patients with SND between those with AVB (Δ9.9 ± 6.3 mmHg vs. Δ9.4 ± 6.3 mmHg, *p* = 0.392) (**Supplementary Table 2**).

**Fig. 4.**
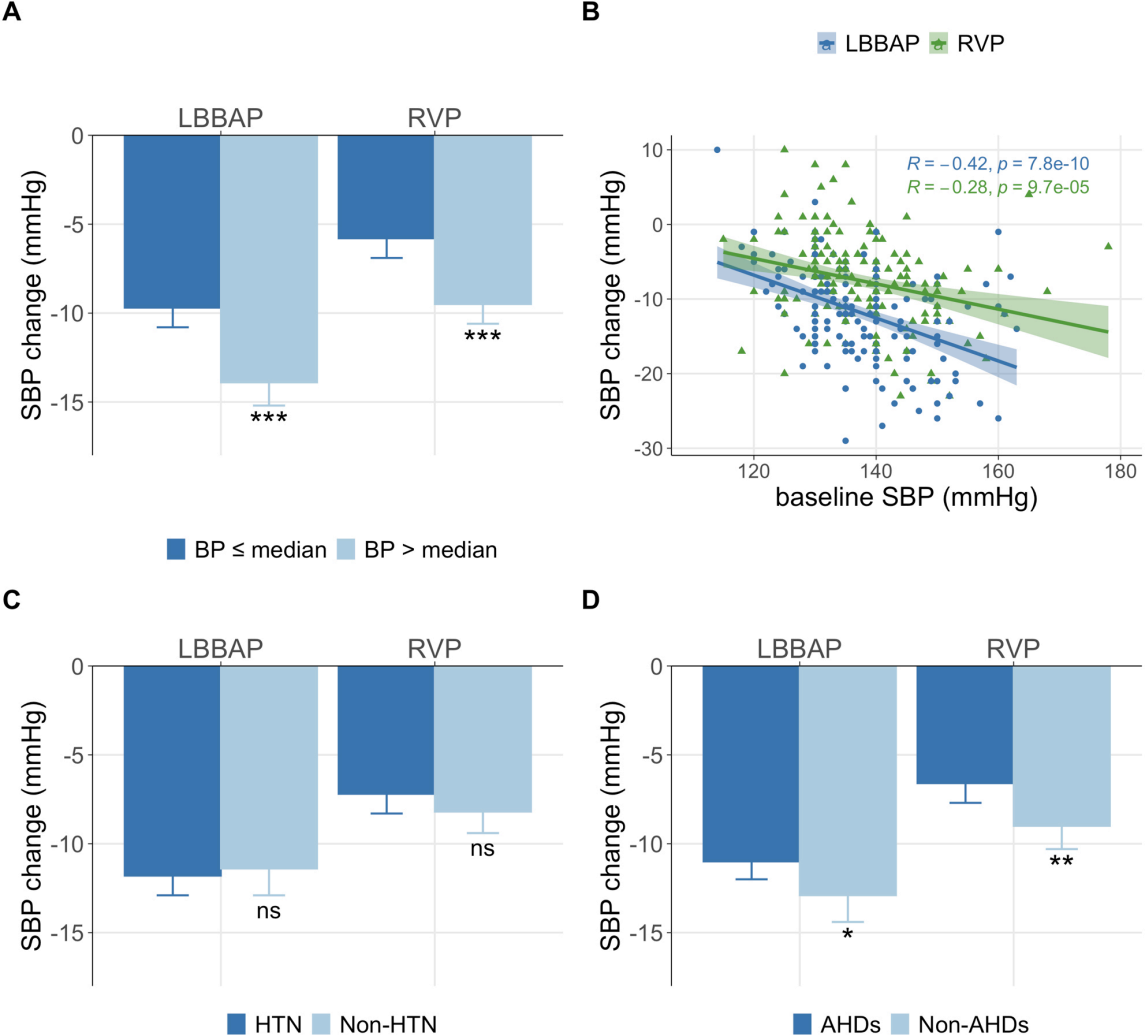
**Association of basic characteristics with SBP change**. (A) SBP change compared between different baseline BP. (B) The correlation between baseline BP and SBP change (R refers to the Pearson correlation coefficient). (C) SBP change compared between patients with or without HTN. (D) SBP change compared between patients with or without AHDs. The error bar referred to the mean ± 95% CI. Notes: The significance levels were presented as follows: * *p* <0.05, ** *p* <0.01, and *** *p* <0.001. Abbreviations: HTN, hypertension; AHDs, anti-hypertensive drugs; BP, blood pressure; LBBAP, left bundle branch area pacing; RVP, right ventricular pacing; SBP, systolic blood pressure; ns, non significant.

Subgroup analyses were conducted in both LBBAP and RVP groups. When assessed by baseline SBP, patients were divided into two groups based on the baseline SBP using the median as a cutoff. Patients with higher baseline SBP had a more pronounced decrease in SBP compared to those with a lower baseline SBP in both the LBBAP and RVP groups (all *p* < 0.001) (Fig. 4A). A significant correlation was observed between baseline SBP and the SBP change post-implantation (*R* = –0.42 for LBBAP and *R* = –0.28 for RVP, both *p* < 0.001) (Fig. 4B). The correlation between the simultaneous HR change and the SBP change post-implantation was displayed in **Supplementary Fig. 1**. There was no significant difference between patients with or without hypertension (HTN) diagnosis in either group (*p* = 0.637 in the LBBAP group and *p* = 0.232 in the RVP group) (Fig. 4C). Patients treated with AHDs had fewer SBP fluctuations than those who did not receive AHDs treatment (*p *= 0.045 in the LBBAP group and *p* = 0.005 in the RVP group) (Fig. 4D). More detailed data on changes in SBP and HR were provided in Table 4. Further subgroup analyses revealed that patients receiving LBBAP exhibited a greater SBP reduction than those receiving RVP who had similar baseline HR and SBP (**Supplementary Table 3**).

**Table 4.** **Subgroup analyses on potential factors influencing SBP change**.

Subgroup		No. of patients	HR, bpm		SBP, mmHg		SBP change, mmHg	*p *value
			Baseline	Acute period	Baseline	Acute period	Mean difference (95% CI)	
LBBAP		193						
Baseline SBP								<0.001
	BP ≤ ${\mathrm{median}}^{a}$	105	56.0 ± 12.6	64.0 ± 5.1	130.2 ± 4.3	120.6 ± 5.3	–9.7 (–10.8, –8.6)	
	BP > median	88	57.7 ± 13.2	66.2 ± 7.1	144.7 ± 6.8	130.7 ± 8.5	–13.9 (–15.2, –12.7)	
Comorbidity								0.637
	HTN	119	55.3 ± 12.9	65.0 ± 6.5	138.6 ± 9.6	126.9 ± 9.4	–11.8 (–12.9, –10.7)	
	Non-HTN	74	59.1 ± 12.5	64.9 ± 5.8	133.9 ± 7.4	122.5 ± 6.1	–11.4 (–12.9, –9.8)	
Drugs								0.045
	AHDs	127	55.3 ± 13.2	65.0 ± 6.2	137.5 ± 9.7	126.5 ± 9.3	–11.0 (–12.0, –9.9)	
	Non-AHDs	66	59.6 ± 11.7	65.0 ± 6.2	135.5 ± 7.7	122.6 ± 6.0	–12.9 (–14.4, –11.3)	
RVP		193						
Baseline SBP								<0.001
	BP ≤ ${\mathrm{median}}^{b}$	100	56.4 ± 12.5	66.3 ± 7.8	130.7 ± 4.2	125.0 ± 6.7	–5.8 (–6.9, –4.7)	
	BP > median	93	59.1 ± 13.4	65.1 ± 6.7	145.2 ± 7.3	135.8 ± 9.0	–9.5 (–10.6, –8.3)	
Comorbidity								0.232
	HTN	115	54.9 ± 12.9	65.2 ± 7.1	139.8 ± 10.0	132.6 ± 9.7	–7.2 (–8.3, –6.1)	
	Non-HTN	78	61.9 ± 11.9	66.5 ± 7.5	134.7 ± 7.3	126.5 ± 8.0	–8.2 (–9.4, –6.9)	
Drugs								0.005
	AHDs	117	55.0 ± 12.9	64.9 ± 6.5	139.3 ± 10.2	132.6 ± 9.8	–6.6 (–7.7, –5.6)	
	Non-AHDs	76	61.9 ± 12.0	66.9 ± 8.3	135.3 ± 7.4	126.3 ± 7.8	–9.0 (–10.3, –7.7)	

^a^ The median SBP of patients in the LBBAP group was 135 mmHg; ^b^ The median SBP of patients in the RVP group was 137 mmHg. Abbreviations: AHDs, antihypertensive drugs; BP, blood pressure; HR, heart rate; HTN, hypertension; LBBAP, left bundle branch area pacing; RVP, right ventricular pacing; SBP, systolic blood pressure.

## 4. Discussion

In this retrospective cohort study with a large sample size, we provided further insight into acute blood pressure changes post-implantation of LBBAP and RVP. Our PS-matched comparison between the LBBAP and RVP group yielded several significant and clinically meaningful findings: (1) the implantation of permanent pacemakers for patients with conduction system disease could contribute to a significant decrease in systolic blood pressure shortly after the implantation; (2) this immediate effect was more pronounced in LBBAP receivers than in RVP receivers, suggesting that LBBAP might have stronger physiological hemodynamic effects; and (3) baseline SBP and the use of anti-hypertensive drugs were potentially associated with the magnitude of arterial BP reduction post pacemaker implantation.

An earlier study noted a potential correlation between the maximal arterial BP differences observed during ventricular pacing, especially comparing AV synchrony with maximal AV asynchrony, and the subsequent improvement in cardiac index [23]. That study suggested that BP variability could be a more valuable indicator of the hemodynamic impact in ventricular-paced individuals. Channon *et al*. [17] reported that paced individuals exhibit a reduction in BP, and the beat-to-beat BP variability was greatly increased when changed from DDD to VVI pacing. A cohort of 24 dual-pacemaker patients focused on the effects of pacing sites on blood pressure, showed that right ventricle (RV) septal pacing was associated with less BP variation compared with RV apical pacing in the VVI mode [15]. Our results with a large study cohort demonstrated an immediate and substantial systolic BP reduction after pacemaker implantation.

Several possible pathophysiological mechanisms may have been responsible for the acute hemodynamic changes in response to pacing. First, almost all patients receiving permanent pacemaker implantation suffer from bradycardias. Compensatory elevation of SBP may occur due to an increased contraction force following increased ventricular filling during bradycardia (the Frank-Starling mechanism), leading to a greater stroke volume (SV) and hence the increased systolic BP [24]. Furthermore, increased left ventricle (LV) filling may stimulate the sympathetic afferent fibers distributed in the heart through cardiac wall distension. Pacing corrects the bradycardia and thus prevents overcompensation of BP. A recent case report [13] suggested that pacemaker implantation resulted in increased cardiac output and a marked reduction in peripheral resistance, which might be due to other less well-understood mechanisms contributing to the decrease in BP.

A similar trend of SBP reduction was observed in both LBBAP and RVP receivers in the present study, however, the magnitude of BP reduction was more profound in the LBBAP group. LBBAP involves placing the ventricular electrode in the left bundle branch region, and our results were consistent with previous studies [25, 26, 27] showing that it produced a narrower paced QRS complex and a relatively lower pacing threshold, leading to improved LV synchrony. In general, improved LV synchrony may increase the stroke volume and cardiac output (CO), which could lead to decreased BP variation. An unexpected trend was observed in the present study. Since the ANS plays an important role in modulating cardiovascular functions and maintaining blood pressure homeostasis [28], we speculated that the ANS might participate in this hemodynamic regulation, since improved cardiac work during LBBAP may cause a decrease in elevated sympathetic activation. RVP is essentially a non-physiological modality that mimics left bundle branch block (LBBB), which may disrupt normal electrical activity and activate the sympathetic nerve system. Therefore, increased sympathetic activity may compensate for the magnitude of BP reduction by pacing. Further research that includes a more comprehensive assessment of ANS and more hemodynamic parameters may prove helpful to evaluate this hypothesis.

Patients with AVB tend to have a higher VP% than those with SND. Our study found that BP variations appeared to be more pronounced in the high-VP% group, which could be attributed to loss of AV synchrony and atrial contraction in patients with a high VP%. However, this does not necessarily imply a direct linear correlation between VP% and BP variations. To date, there is no evidence suggesting a dose-dependent correlation between BP reduction and pacing burden. In patients with a low VP% (e.g., VP% ≤1%), atrial pacing may still cause BP variations, yet its influence may be less than ventricular pacing. Nevertheless, we did not find a significant difference in SBP between patients with AVB and those with SND. In fact, several confounding factors related to conduction system dysfunction (baseline BP, atrioventricular conduction, ANS modulation), might play a more important role in blood pressure fluctuations and could have influenced the combined results. Therefore, these factors should be comprehensively considered when interpreting our findings, and further research is needed to determine the exact association between BP reduction and VP%.

The regulation of blood pressure in humans is a highly intricate process. In the present study, we tried to identify which subgroup would experience more pronounced BP variations. A significant difference in SBP change was found between patients grouped by their baseline SBP and those grouped by the use of AHDs, while changes were not comparable between patients grouped by their combined history of hypertension. Multiple studies have clearly illustrated that abnormal sympathetic activity may be responsible for the appearance and maintenance of high blood pressure [29, 30, 31]. In our study, the mean baseline SBP was around 145 mmHg in the group with a higher baseline SBP, meeting the diagnostic criteria for hypertension [20]. Instantaneous sympathetic tone may be higher in these patients. In contrast, certain anti-hypertensive medications may reduce sympathetic activity and improve vagal cardiac control [32, 33], potentially resulting in a relatively lower basal sympathetic tone in patients treated with these drugs compared to those without AHDs. Conversely, patients with a history of hypertension, who have undergone long-term anti-hypertensive treatments, may no longer differ from those without a HTN history in baseline BP and sympathetic tone. This could explain why higher baseline BP was potentially linked to high BP variation while the use of AHDs produced an opposite trend. Based on these findings, we assumed that higher baseline sympathetic activity may contribute to a greater magnitude of SBP reduction after the initiation of pacing therapy that alleviates sympathetic activity. No relevant studies are currently available, and future experimental and clinical investigations are necessary to validate our assumption.

As a new emerging pacing strategy, LBBAP delivers a huge breakthrough in conduction system pacing, and it is meaningful to illustrate its hemodynamic outcomes. This study produced a preliminary insight into the acute variations in blood pressure during pacemaker implantation and the initiation of pacing therapy by comparing LBBAP with RVP using propensity score matching. Our findings will help clinicians gain a comprehensive understanding of the LBBAP technique and promote its further adoption. It is worth noting that the acute hemodynamic responses may differ between *de-novo* pacemaker recipients and chronically paced patients, highlighting the requirement for further research on its long-term hemodynamic impact. Moreover, it is necessary for future prospective studies to comprehensively measure and record more hemodynamic parameters such as cardiac output, total peripheral resistance, and respiratory rate, and characterize the timeline of hemodynamic changes during pacing therapy. Since LBBAP produces a more synchronized ventricular motion, future studies could also incorporate echocardiographic assessment to evaluate the relationship between hemodynamic changes and improved cardiac function.

## 5. Limitations

The present study has several limitations. First, it was a single-center retrospective analysis, and the potential bias inherent in a non-randomized design cannot be avoided. Although we performed PSM to minimize the confounding factors between LBBAP and RVP patients, heterogeneity among patients still remained. Second, blood pressure is a dynamic parameter that can be influenced by various factors. Specifically, some of the enrolled patients receiving pacemaker implantation were critically ill. During critical illness, the body undergoes significant stress and physiological changes that can affect blood pressure. Inpatient hospital stays can cause BP fluctuations for other hospitalized patients, stemming from factors such as stress, medications, fluid imbalances, and illness progression. In contrast to home/ambulatory blood pressure, measuring blood pressure during inpatient stays may offer a less comprehensive assessment. This limitation can interfere with our ability to accurately reflect the true BP changes. Third, the use of an arm pressure cuff for BP measurement could have introduced some measurement errors. As this study was conducted retrospectively, it was not feasible to utilize invasive blood pressure transducers to confirm the accuracy of external arm cuff-based pressure readings for each participant. Additionally, the majority of enrolled patients had no indications for invasive blood pressure monitoring. In the future, we can consider applying invasive blood pressure monitoring techniques to observe the effects of pacing on blood pressure in appropriate patients. Fourth, the present study did not investigate the physiological mechanisms underlying the acute BP reduction caused by pacing. We speculate that pacing may have corrected the bradycardia and thereby eliminated the associated compensatory BP elevation. We considered this phenomenon as a physiological response, returning the blood pressure to the patient’s normal level. However, it is essential to note that significant periprocedural BP fluctuations could increase the risk of adverse events. Therefore, close monitoring of blood pressure should be implemented during the periprocedural period of pacing. Future well-designed clinical or mechanical studies could simultaneously measure other hemodynamic parameters to gain deeper insights into the mechanisms underlying this phenomenon.

## 6. Conclusions

Permanent pacemaker implantation may contribute to a decrease in systolic blood pressure shortly after implantation, which is more prominent in LBBAP receivers. Baseline SBP and the use of anti-hypertensive drugs are potentially associated with the magnitude of BP variation. Further large-scale prospective studies are needed to confirm the exact relationship between cardiac pacing and blood pressure variation, as well as the long-term hemodynamic effects of LBBAP versus RVP.

## Data Availability

The original data presented in the study are included in the article, further inquiries can be directed to the corresponding authors.

## Abbreviations

ACEi/ARB, angiotensin-converting enzyme inhibitor /angiotensin receptor blocker; AF/AFL, atrial fibrillation/atrial flutter; AHDs, antihypertensive drugs; ANS, autonomic nervous system; AVB, atrioventricular block; BP, blood pressure; CCB, calcium channel blockers; CO, cardiac output; DBP, diastolic blood pressure; ECG, electrocardiogram; HR, heart rate; HTN, hypertension; IVS, interventricular septum; LBBAP, left bundle branch area pacing; LVAT, left ventricular activation time; LVEDD, left ventricular end-diastolic dimension; LVEF, left ventricular ejection fraction; PSM, propensity score-matching; RBBD, right bundle branch conduction delay; RVP, right ventricular pacing; SBP, systolic blood pressure; SD, standard deviation; SND, sinus node dysfunction.

## Supplementary Material

Supplementary material associated with this article can be found, in the online version, at https://doi.org/10.31083/j.rcm2412372.
