# Supplementary material for: Left Bundle Branch Area Pacing Contributes to a Greater Acute Blood Pressure Reduction Compared to Right Ventricular Pacing
Source: Rev Cardiovasc Med. 2023 Dec 27;24(12):372. doi: 10.31083/j.rcm2412372 (PMC11272860; doi:10.31083/j.rcm2412372)
Supplement: Supplementary file 1 [file 2153-8174-24-12-372-s1.zip › 2153-8174-24-12-372-s1.pdf]

Supplementary Material

Supplementary Table 1. Patients characteristics across different VP%.

	VP% ≤ 1%	1% < VP% ≤ 20%	20% < VP% ≤ 40%	VP% > 40%
HR, bpm	52.6 ± 10.3	56.7 ± 12.4	57.6 ± 12.8	59.3 ± 13.8
AVB, n	27/60	54/109	30/64	111/153
AVB, %	45.0%	49.5%	46.9%	72.5%
AP, %	43.0 ± 41.4	35.8 ± 40.1	44.4 ± 36.5	25.6 ± 31.6

Abbreviations: AP, atrial pacing; AVB, atrioventricular block; HR, heart rate; VP%, percentage of ventricular pacing.

Supplementary Table 2. SBP changes comparing between indications.

	SND	AVB	*P* value
n	164	222	
HR, bpm	58.4 ± 11.7	56.4 ± 13.6	0.115
Programmed lower rate, bpm	59.9 ± 0.8	59.9 ± 0.7	0.934
VP,%	14.2(1.1, 42.0)	40.2(9.4, 99.9)	< 0.001
ΔSBP, mmHg	9.9 ± 6.3	9.4 ± 6.3	0.392

Abbreviations: AVB, atrioventricular block; SND, sinus node dysfunction

Supplementary Table 3. SBP changes comparing between LBBAP and RVP receivers who had similar baseline HR and baseline SBP.

	LBBAP				RVP					
Group	baseline HR	baseline SBP	SBP at acute period	ΔSBP	baseline HR	baseline SBP	SBP at acute period	ΔSBP	*P* for SBP change	95%CI
HR > median + BP > median	68.4 ± 8.0	143.3 ± 5.8	126.1 ± 5.0	17.3 ± 4.8	69.4 ± 8.1	143.8 ± 6.0	131.5 ± 6.0	12.4 ± 4.7	< 0.001	[3.0, 6.8]
HR > median + BP ≤ median	67.7 ± 8.0	131.3 ± 3.5	118.7 ± 5.3	12.6 ± 5.6	67.2 ± 7.4	130.3 ± 4.1	122.0 ± 6.7	8.3 ± 5.2	< 0.001	[2.0, 6.6]
HR ≤ median + BP > median	46.5 ± 6.4	146.0 ± 7.6	135.5 ± 8.7	10.5 ± 5.2	47.0 ± 5.7	145.4 ± 8.9	140.1 ± 9.4	5.3 ± 3.4	< 0.001	[3.3, 7.0]
HR ≤ median + BP ≤ median	46.8 ± 6.1	129.4 ± 4.6	122.0 ± 4.9	7.4 ± 4.5	46.7 ± 6.7	130.2 ± 4.0	126.4 ± 6.0	3.7 ± 5.0	< 0.001	[1.9, 5.5]

Notes: The median baseline SBP of the overall patients was 135mmHg and the median baseline HR was 56bpm. ΔSBP refers to the change in SBP.
Abbreviations: HR, heart rate; LBBAP, left bundle branch pacing; RVP, right ventricular pacing; SBP, systolic blood pressure.

Supplementary Fig. 1. The correlation between HR change and SBP change.

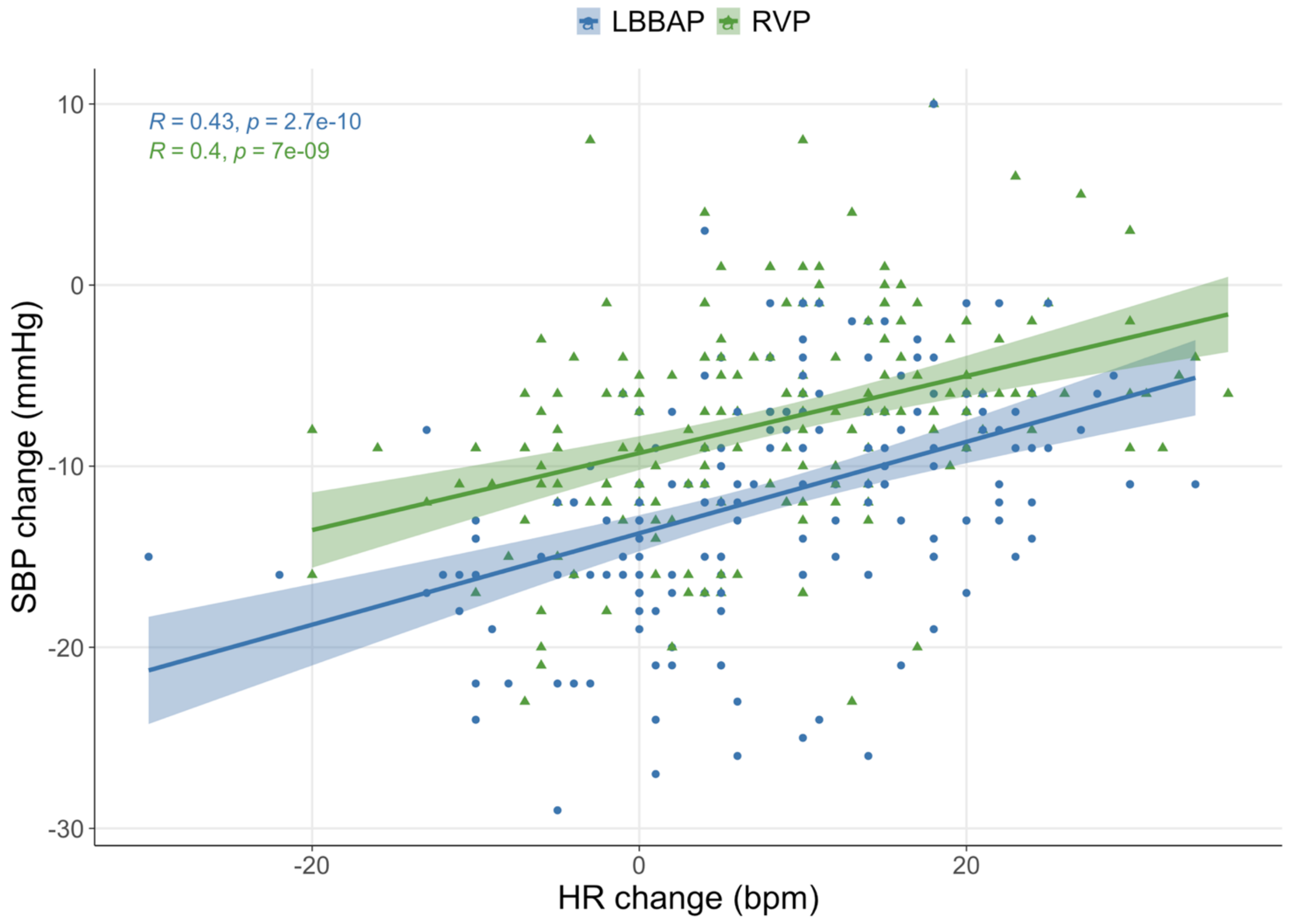

Notes: HR change refers to the difference between baseline HR and HR at the acute period. *R* refers to the Pearson correlation coefficient.

Abbreviations were the same as Supplementary Table 3.
